# Urinary apolipoprotein A4 as a biomarker for renal allograft injury in kidney transplant recipients

**DOI:** 10.1371/journal.pone.0324529

**Published:** 2025-05-21

**Authors:** Youn Kyung Kee, Dong Ho Shin, Jieun Oh, Ji Young Park, Dong Hyun Kim, Kyungjai Ko, Samuel Lee, Hee Jung Jeon

**Affiliations:** 1 Department of Internal Medicine, Kangdong Sacred Heart Hospital, Hallym University College of Medicine, Seoul, Republic of Korea; 2 Department of Laboratory Medicine, Kangdong Sacred Heart Hospital, Hallym University College of Medicine, Seoul, Republic of Koreas; 3 Department of Surgery, Kangdong Sacred Heart Hospital, Hallym University College of Medicine, Seoul, Republic of Korea; 4 Department of Surgery, Kangnam Sacred Heart Hospital, Hallym University College of Medicine, Seoul, Republic of Korea; University of Montenegro-Faculty of Medicine, MONTENEGRO

## Abstract

Chronic renal allograft injury (CRAI) is a major cause of allograft loss in kidney transplant recipients (KTRs). The aim of this study was to evaluate the associations of urinary apolipoprotein A4 (ApoA-IV) levels with renal function and rapid renal function decline in KTRs. This study included 50 KTRs. Proteomic analysis via liquid chromatography‒mass spectrometry and tandem mass spectrometry (LC-MS/MS) was performed to identify potential urinary biomarkers. The SWATH (sequential window acquisition of all theoretical mass spectra) method was used for protein quantification. Urinary ApoA-IV levels were validated by enzyme-linked immunosorbent assay (ELISA). Rapid renal function decline was defined as an estimated glomerular filtration rate (GFR) decrease of >3 mL/min/1.73 m^2^ per year or initiation of dialysis. The log-transformed urinary ApoA-IV levels measured by ELISA had a significantly inverse correlation with the estimated GFR (r = -0.72, *P* < 0.001). Moreover, urinary ApoA-IV levels were higher in patients with rapid renal function decline than in those with stable renal function (215.4 ± 181.8 μg/mL vs. 42.5 ± 72.4 μg/mL, *P* = 0.001). Univariate logistic regression analysis revealed that log-transformed urinary ApoA-IV levels were significantly associated with rapid renal function decline (odds ratio [OR] 6.70, 95% confidence interval [CI] 2.56–22.83; *P* < 0.001). Multiple logistic regression showed urinary ApoA-IV levels remained a significant risk factor for rapid renal function decline (OR 4.10, 95% CI 1.10–19.55; *P* = 0.047). ROC curve analysis revealed the area under the curve (AUC) of 0.834 (95% CI 0.722–0.945, *P* < 0.001) for urinary ApoA-IV levels in predicting rapid renal function decline. Our results suggest that urinary ApoA-IV levels might be a potential biomarker for renal allograft function and could be used as a predictor for rapid renal function decline in KTRs.

## Introduction

Although the short-term outcomes of kidney transplantation have improved remarkably over several decades with the introduction of potent immunosuppression, the long-term allograft survival rates of kidney transplantation remain similar [1]. Chronic renal allograft injury (CRAI) is a major cause of allograft loss in kidney transplant recipients (KTRs). The CRAI is a multifactorial clinical and pathological entity characterized by a progressive decline in the glomerular filtration rate (GFR), which is generally associated with several immunological and nonimmunological factors [2,3]. Immunological factors associated with the CRAI include sensitization, cellular and/or antibody-mediated rejection, and HLA mismatch, and nonimmunological factors include delayed graft function, recurrent infection episodes, arterial hypertension, recurrent glomerulonephritis, poor-quality donor kidney, ureteral stenosis, and calcineurin inhibitor nephrotoxicity. Histologically, CRAI is characterized by interstitial fibrosis and tubular atrophy [4].

Apolipoprotein A4 (ApoA-IV), which is known to have anti-atherogenic properties, is also known to be elevated in patients with chronic kidney disease [5]. In addition, several previous studies have reported that the serum ApoA-IV level could predict the progression of renal impairment in patients with nondiabetic primary kidney disease as well as in patients with type 2 diabetes mellitus (DM) [6–8]. Recent years have seen growing interest in identifying non-invasive urinary biomarkers for monitoring renal allograft function and predicting outcomes in KTRs. Several promising candidates have emerged, including neutrophil gelatinase-associated lipocalin (NGAL), kidney injury molecule-1 (KIM-1), and β2-microglobulin [9]. These biomarkers have shown potential in detecting acute kidney injury and predicting graft function, but their utility in CRAI remains under investigation [10].

Based on previous studies showing the association between serum ApoA-IV levels and renal function decline in various kidney diseases, we hypothesized that urinary ApoA-IV levels could serve as a potential biomarker for renal allograft function and predictor of rapid renal function decline in KTRs. We aimed to evaluate the associations between urinary ApoA-IV levels and both current renal function and future renal function decline in KTRs.

## Materials and Methods

### Ethics statement

The Institutional Review Board of Kangdong Sacred Heart Hospital approved this study (IRB no. 2015-07-006-001), which was conducted in accordance with the Declaration of Helsinki principles and the Istanbul Declaration. All participants provided written informed consent prior to the start of this study.

### Selection of study subjects

A total of fifty KTRs who underwent follow-up at the Kangdong Sacred Heart Hospital transplant clinic between 30 November 2015 and 7 January 2016 were included in this study. The inclusion criteria were adult patients over 18 years of age who had undergone kidney transplantation for more than one year and who provided informed consent for this study. This study excluded patients on dialysis due to graft failure, acute rejection within 3 months, and active infections, including urinary tract infections. Patients on dialysis due to graft failure were excluded because their urinary protein profiles would be significantly altered and not representative of functioning kidney transplants. Patients with acute rejection within 3 months or active infections were excluded to avoid confounding effects on urinary protein levels that might not reflect CRAI. Patients were divided into two groups: a control group (n = 20) with normal renal function and a CRAI group (n = 30) with an estimated GFR of less than 60 mL/min/1.73 m2. Rapid renal function decline was defined as a decrease in the estimated GFR of more than 3 mL/min/1.73 m^2^ per year or initiation of dialysis. The definition of rapid renal function decline was referred to previous studies [11–13].

### Sample preparation and proteomic analysis

Urine samples were collected from the enrolled KTRs and stored in a refrigerator at −70°C until proteomic analysis. Prior to proteomic analysis, samples were centrifuged at 3,300 × g for two minutes. The modified trypsin (Promega, USA) was used to digest the protein from the urine samples at 37°C overnight, and the peptides were recovered using a C18 cartridge (Sep-Pak, Waters, USA) and completely dried in a speed-vacuum. Proteomic analysis via liquid chromatography‒mass spectrometry and tandem mass spectrometry (LC‒MS/MS) was carried out via Triple-TOF-TM 5600+ (AB Sciex, Canada). The peptide separation was achieved using an Eksigent NanoLC-2D+ with Nanoflex cHiPLC system. Peptide samples were loaded at 1 μL/min onto a trap column (0.5 mm × 200 μm) and separated on an analytical column (15 cm × 75 μm) using a 2% to 35% gradient of acetonitrile with 0.1% formic acid over 30 minutes at a flow rate of 400 nL/min, followed by column regeneration by washing with 60% solvent for 50 min and equilibrating with 2% solvent for 10 min. For data-dependent acquisition, Triple-TOF-TM 5600 + mass spectrometer performed a 50-ms survey scan and a 50-ms automated MS/MS scan on the 15 most intense ions, with a precursor intensity of 150 counts, a charge state above 1, and a dynamic exclusion time option of 6 seconds to avoid repeated scans of the same ion. Ions were isolated at 0.7 Da resolution and fragmented with collision energy ramped from 15 to 45 eV over 50 ms for optimal fragmentation. The SWATH (sequential window acquisition of all theoretical mass spectra) mass spectrometry was used to ensure a comprehensive analysis, operating in looped product ion mode across 20 Da windows with a 1 Da overlap within the 400–1000 Da range. Each SWATH window received a customized collision energy for optimal fragmentation of doubly charged ions, and an accumulation time of 80 ms per fragment ion scan enabled high sensitivity across the entire duty cycle. This setup is designed to maximize peptide identification while also being sensitive to detect low-abundance proteins, providing a robust platform for deep proteome profiling. The SWATH approach is straightforward, reliable, and highly reproducible for proteomic analysis; thus, it could be applied to characterize proteomic analyses in large numbers of samples [14–16].

### Verification by ELISA and data analysis

Through the SWATH method, the top 96 most highly expressed proteins were determined, along with quantification information for each protein after normalized to total protein abundances. These data were then compared between the CRAI group and the control group. The levels of several urinary proteins, including ApoA-IV, RBP4, SERPINF1, CA1, and B2M, were significantly greater in the CRAI group than in the control group. The list of 96 highly expressed proteins and the results of principal component analysis and volcano plot are shown in S1 Table, S1 and S2 Figures in S1 File. To verify the significant protein levels, including those of ApoA-IV, we conducted an enzyme-linked immunosorbent assay (ELISA) using the same urine samples. ELISA for ApoA-IV was performed using a commercial kit (Human ApoA4 ELISA Kit, Elabscience) according to the manufacturer’s instructions. A standard curve was generated for each assay using known concentrations of recombinant human ApoA-IV. The estimated GFR was calculated via the modification of diet in the renal disease study equation [17].

### Statistical analysis

Continuous values are expressed as means ± standard deviations or median [interquartile range] as per Shapiro-Wilk normality test. Categorical values are expressed as frequencies (percentages) in the descriptive analysis. As appropriate, the chi-square test or the Fisher’s exact test was used to compare categorical data between groups, and Student’s t test was used to compare continuous variables. Urinary ApoA-IV levels and renal function were analyzed via Pearson correlation, and factors associated with rapid renal function decline in KTRs were analyzed via univariate and multiple logistic regression. The covariates selected for the multiple logistic regression model were chosen based on their established clinical relevance to renal allograft function and outcomes. Urinary ApoA-IV levels were also analyzed via a receiver operating characteristic (ROC) curve to determine its correlation with rapid decline in renal function. The optimal cut-off value in ROC curve analysis was determined by Youden’s J Statistic [18]. For the statistical analyses, SPSS version 18.0.0 (SPSS Inc., Chicago, IL) and R version 4.0.1 (www.r-project.org; The R Foundation for Statistical Computing, Vienna, Austria) software packages were utilized. A *P* value of less than 0.05 was considered significant.

## Results

### Demographic and clinical characteristics of the study subjects

In this study, 62.0% of the recipients were male, and the mean age at transplantation was 39.5 ± 10.3 years. The recipients’ mean estimated GFR and serum creatinine levels were 62.9 ± 25.8 mL/min/1.73 m^2^ and 1.3 ± 0.6 mg/dL, respectively, at the time of sampling. At transplantation, the mean age of the donors was 35.8 ± 15.7 years, with 70.0% being male. Table 1 shows a summary of the demographic and clinical characteristics of the subjects.

**Table 1:** Demographic and clinical characteristics of the study subjects.

Parameters	Chronic renal allograft injury group (n = 30)	Normal renal function group (n = 20)	Total	*P* value ^a^
Age at transplantation (year)	37.3 ± 10.1	42.8 ± 9.9	39.5 ± 10.3	0.065
Sex (%, male)	20 (66.7%)	11 (55.0%)	31 (62.0%)	0.592
Diabetes (%) ^b^	9 (30.0%)	5 (25.0%)	14 (28.0%)	0.949
Pretransplant dialysis (%)				0.342
- Hemodialysis	20 (66.7%)	12 (60.0%)	32 (64.0%)	
- Peritoneal dialysis	7 (23.3%)	7 (35.0%)	14 (28.0%)	
- Preemptive	3 (10.0%)	1 (5.0%)	4 (8.0%)	
Duration of dialysis (month)	48.0 [22.5–84.0]	32.0 [12.0–64.5]	39.0 [13.0–72.0]	0.315
Cause of ESRD (%)				0.244
- Diabetes	3 (10.0%)	2 (10.0%)	5 (10.0%)	
- Glomerulonephritis	10 (33.3%)	2 (10.0%)	12 (24.0%)	
- Hypertension	3 (10.0%)	2 (10.0%)	5 (10.0%)	
- Others	1 (3.3%)	4 (20.0%)	5 (10.0%)	
- Unknown	13 (43.3%)	10 (50.0%)	24 (46.0%)	
Months after transplantation at sampling (month)	164.8 [102.1-229.2]	147.9 [64.7-208.2]	164.8 [81.0-222.4]	0.151
Serum creatinine at sampling (mg/dL)	1.6 [1.4-1.7]	0.8 [0.7-1.0]	1.3 [0.9-1.7]	<0.001
Estimated GFR at sampling (mL/min/1.73m^2^)	44.0 ± 10.5	91.3 ± 11.7	62.9 ± 25.8	<0.001
Calcineurin inhibitor (%)				0.434
- Tacrolimus	9 (30.0%)	9 (45.0%)	18 (36.0%)	
- Cyclosporine	21 (70.0%)	11 (55.0%)	32 (64.0%)	
HLA mismatch (number)	3.3 ± 1.3	3.1 ± 1.4	3.2 ± 1.3	0.636
Donor type (%)				0.513
- Living donor	13 (43.3%)	6 (30.0%)	19 (38.0%)	
- Deceased donor	17 (56.7%)	14 (70.0%)	31 (62.0%)	
Acute rejection episode (%)	11 (36.7%)	0 (0.0%)	11 (22.0%)	0.002
Donor age at transplantation (year)	36.5 ± 16.1	34.8 ± 15.3	35.8 ± 15.7	0.716
Donor sex (%, male)	19 (63.3%)	16 (80.0%)	35 (70.0%)	0.345

Continuous values are expressed as means ± standard deviations or median [interquartile range] as per Shapiro-Wilk normality test. Categorical values are expressed as frequencies (percentages). ^a^ Continuous variables were compared via t tests, and categorical variables were compared via the chi-square test or the Fisher’s exact test, as appropriate.

^b^Diabetes includes new-onset diabetes after transplantation

Conversion factors for units: serum creatinine in mg/dL to μmol/L, × 88.4.

ESRD, end-stage renal disease; GFR, glomerular filtration rate; HLA, human leukocyte antigen.

### Association between urinary ApoA-IV and renal function in kidney transplant patients

**Fig 1:**
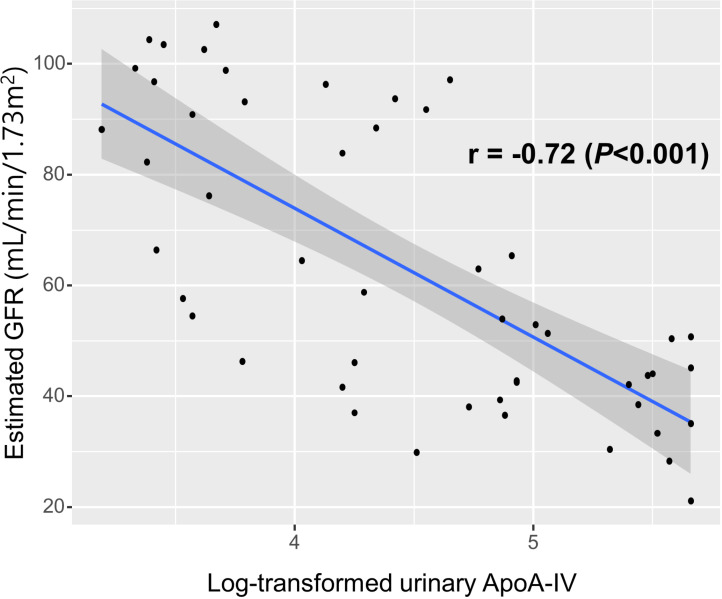
The correlation between urinary apolipoprotein A4 (ApoA-IV) levels and renal allograft function according to estimated glomerular filtration rate in kidney transplant recipients. The log-transformed urinary ApoA-IV levels measured by ELISA had significantly inverse correlation with the estimated glomerular filtration rate in kidney transplant recipients.

The levels of urinary ApoA-IV were inversely correlated with renal function in KTRs. The log-transformed urinary ApoA-IV levels measured by ELISA had a significantly inverse correlation with the estimated GFR at the time of initial sampling in KTRs (r = -0.72, *P* < 0.001) (Fig 1). In addition, among the 50 subjects, urinary ApoA-IV levels were significantly higher in the CRAI group than in the control group (170.9 ± 166.3 μg/mL vs. 14.2 ± 20.0 μg/mL, *P* < 0.001).

### Association between urinary ApoA-IV and rapid renal function decline in kidney transplant patients

**Fig 2:**
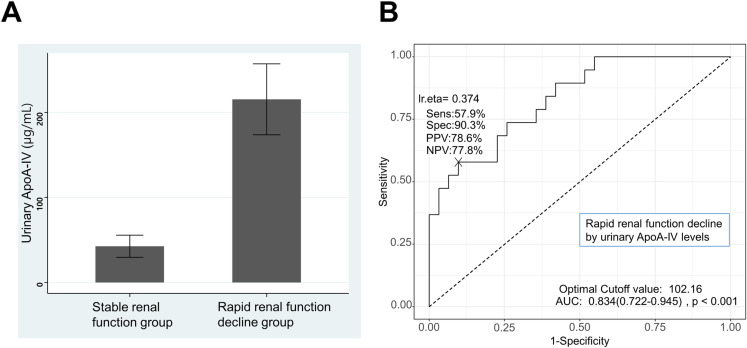
Association between urinary apolipoprotein A4 (ApoA-IV) levels and rapid renal function decline in kidney transplant recipients. **(A)** Comparison of urinary ApoA-IV levels between the rapid renal function decline group and the stable renal function group in kidney transplant recipients. Boxplot showing significantly higher urinary ApoA-IV levels in patients with rapid renal function decline. **(B)** ROC curve of ApoA-IV for rapid renal function decline in kidney transplant recipients. ROC curve demonstrating the predictive value of urinary ApoA-IV for rapid renal function decline (AUC = 0.834). Urinary ApoA-IV levels above 102.16 μg/mL were associated with increased risk of rapid renal function decline.

Among the 50 KTRs, 19 (38%) met the criteria for rapid renal function decline. Urinary ApoA-IV levels measured by ELISA were greater in patients with rapid renal function decline than in those with stable renal function (215.4 ± 181.8 μg/mL vs. 42.5 ± 72.4 μg/mL, *P* = 0.001) (Fig 2A). Univariate logistic regression analysis revealed that log-transformed urinary ApoA-IV levels were also significantly associated with rapid renal function decline (odds ratio [OR] 6.70, 95% confidence interval [CI] 2.56–22.83; *P* < 0.001). In the multiple logistic regression adjusted for recipient age, recipient sex, donor age, number of HLA mismatches, acute rejection episodes, and CRAI, urinary ApoA-IV levels remained a significant risk factor for rapid renal function decline (OR 4.10, 95% CI 1.10–19.55; *P* = 0.047) (Table 2). These results align with our initial hypothesis that urinary ApoA-IV levels would be associated with rapid renal function decline in KTRs. The significant odds ratio in the multiple logistic regression analysis supports the potential of urinary ApoA-IV as an independent predictor of renal function decline. We also performed simple and multiple linear regression analysis for factors related to annual declines of estimated GFR (mL/min/1.73m2 per year), and urinary ApoA-IV levels were significantly related to annual declines of estimated GFR (S2 Table in S1 File). In addition, the ROC curve analysis revealed that the area under the curve (AUC) of urinary ApoA-IV levels for rapid renal function decline was 0.834 (95% CI 0.722–0.945, *P* < 0.001) (Fig 2B). The optimal cut-off value of 102.16 μg/mL for urinary ApoA-IV was determined using Youden’s J Statistic, which maximizes the sum of sensitivity and specificity. This method provides an objective criterion for selecting the most appropriate threshold in ROC curve analysis [18]. With the 102.16 μg/mL cut-off value, the positive and negative predictive values were 78.6% and 77.8% for predicting rapid renal function decline, respectively. When the patients were stratified into two groups by the cut-off value (102.16 μg/mL) of urinary ApoA-IV levels, the upper group also had more rapid renal function decline (76.9% vs. 24.3%) and graft failure (23.1% vs. 5.4%) than did the lower group.

**Table 2:** Logistic regression analysis of factors associated with rapid renal function decline in kidney transplant recipients.

Variables	Univariate logistic regression			Multiple logistic regression ^a^		
	OR	95% CI	*P* value	OR	95% CI	*P* value
Recipient male sex (vs. female)	2.31	0.69-8.59	0.187	2.93	0.49-22.14	0.255
Recipient age	0.96	0.90-1.02	0.169	1.01	0.92-1.13	0.767
Donor age	0.99	0.95-1.02	0.463	0.97	0.90-1.03	0.295
Number of HLA mismatches	1.19	0.76-1.92	0.461	1.22	0.67-2.43	0.529
Acute rejection episodes	2.40	0.61-9.81	0.207	1.17	0.17-9.47	0.878
Chronic renal allograft injury ^b^	28.50	4.90-546.9	0.002	10.26	0.99-246.99	0.073
Urinary ApoA-IV (log-transformed)	6.70	2.56-22.83	<0.001	4.10	1.10-19.55	0.047

^a^ Adjusted for recipient age, recipient sex, donor age, number of HLA mismatches, acute rejection episodes, and chronic renal allograft injury.

^b^ Chronic renal allograft injury was defined as an estimated GFR of less than 60 mL/min/1.73 m2.

OR, odds ratio; CI, confidence interval; HLA, human leukocyte antigen; ApoA-IV, apolipoprotein A4.

## Discussion

In our study, the levels of urinary ApoA-IV were inversely correlated with renal allograft function in KTRs. Urinary ApoA-IV levels were significantly higher in the CRAI group than in the control group. Moreover, urinary ApoA-IV levels were also higher in patients with rapid renal function decline than in those with stable renal function and remained a significant factor for rapid renal function decline according to logistic regression analysis. The ROC curve analysis showed an AUC of 0.834, indicating good discriminatory ability. At the cut-off value of 102.16 μg/mL, the positive and negative predictive values were 78.6% and 77.8%, respectively. It suggests the clinical utility of ApoA-IV as a predictive biomarker for the risk of rapid renal function decline in KTRs.

ApoA-IV, a 46 kDa glycoprotein, is an apolipoprotein that is synthesized mainly in small intestinal enterocytes and is released as one of the structural proteins of chylomicrons, high-density lipoproteins, and very low-density lipoproteins, or it can be detected in its free form in plasma [19–21]. In vitro studies have indicated that ApoA-IV plays a role in reverse cholesterol transport by activating lipoprotein lipase, lecithin-cholesterol acyltransferase, and cholesteryl ester transfer protein, resulting in anti-atherogenic properties [22–25]. Studies in mice overexpressing human or mouse ApoA-IV revealed fewer atherosclerotic aortic lesions than in control mice [26,27]. Additionally, in human studies, lower levels of plasma ApoA-IV were found in patients with coronary heart disease than in control subjects [28,29].

In addition, plasma ApoA-IV levels are also known to be elevated in patients with chronic kidney disease or renal failure. High levels of plasma ApoA-IV are strongly associated with a low estimated GFR and are suggested as early markers of impaired kidney function [5,30,31]. Moreover, Boes E et al. [6] reported that ApoA-IV predicts the progression of chronic kidney disease in patients with nondiabetic primary kidney disease, and Cheng CW et al. [7] and Peters KE et al. [8] presented the role of serum ApoA-IV in predicting a rapid decline in renal function in patients with type 2 DM. The results of those studies are consistent with our findings that urinary ApoA-IV levels are associated with decreased renal function and could be a potential biomarker for rapid decline in renal allograft function in KTRs. The urinary ApoA-IV concentration remained a significant factor for rapid renal function decline in the multiple logistic regression analysis adjusted for multiple covariates, including the CRAI. ApoA-IV is filtered by the glomeruli and reabsorbed by proximal tubular cells, as shown by an immunohistochemical analysis of healthy human kidney tissue samples and studies of urine ApoA-IV in patients with proteinuria or Dent’s disease [32,33]. However, it is still unclear whether the increased levels of ApoA-IV in renal disease patients are only due to their impaired ability to filter or whether ApoA-IV is also involved in a defense mechanism against the disease [30].

While our study demonstrates a correlation between urinary ApoA-IV levels and renal allograft function, the specific mechanisms linking ApoA-IV to CRAI and long-term transplant outcomes remain to be fully elucidated. However, the elevated levels we observed in patients with declining renal function suggest that urinary ApoA-IV may also be a biomarker of ongoing allograft injury. It could allow for earlier identification of patients at risk of allograft dysfunction, enabling more timely interventions. Moreover, as a non-invasive test, it could reduce the need for frequent biopsies, thereby decreasing patient discomfort and potential complications. Our findings align with recent research on novel biomarkers for renal function decline. Peters et al. [8] identified several circulating biomarkers, including ApoA-IV, which predicted rapid decline in renal function in patients with type 2 diabetes. Their study, like ours, highlights the potential of ApoA-IV as a predictive biomarker for renal outcomes, suggesting its relevance across different patient populations with kidney disease. While our findings suggest that urinary ApoA-IV is a promising biomarker for CRAI, the pathophysiological mechanisms by which ApoA-IV influences renal function remain unclear. It is essential to determine whether ApoA-IV plays a causal role in renal dysfunction or merely serves as a marker of disease progression in future mechanistic studies.

While our study demonstrates the potential of urinary ApoA-IV as a biomarker for renal allograft function and predictor of rapid renal function decline, it is important to contextualize these findings with other established urinary biomarkers such as NGAL and KIM-1. Previous studies have shown that urinary NGAL and KIM-1 are effective in detecting acute kidney injury and predicting short-term graft function in KTRs [34–39]. Kielar et al. [35] reported that urinary NGAL measured after 1 year post-transplant predicts the relative and absolute changes in estimated GFR during the follow-up in 109 KTRs. The AUC of urinary NGAL in the prediction of >10% decrease in estimated GFR was 0.645 (95% CI 0.529–0.760) in ROC curve analysis [35]. Zhu et al. [39] assessed the predictive utility of urinary KIM-1 levels in 160 KTRs and found that elevated urinary KIM-1 on the first day after transplantation was associated with a 23.5% higher risk of delayed graft function and a 27.3% greater risk of prolonged renal allograft dysfunction. However, their utility in predicting long-term outcomes and chronic allograft nephropathy remains under investigation. While ApoA-IV is primarily synthesized in the intestine and filtered by the glomeruli, with altered levels potentially reflecting both systemic metabolic changes and kidney function [40], NGAL and KIM-1 reflect renal tubular cell injury so that they rise rapidly following acute kidney injury and remain elevated in chronic kidney disease [41,42]. Our findings suggest that urinary ApoA-IV may complement existing biomarkers by providing additional information on metabolic and inflammatory processes affecting the allograft. Combining ApoA-IV with NGAL and KIM-1 could offer a more comprehensive assessment of allograft health, particularly in the context of CRAI and long-term renal function decline. Further studies directly comparing the performance of ApoA-IV with NGAL and KIM-1 in the same cohort of KTRs are needed to fully elucidate their relative clinical utility. Such comparisons should assess not only their individual predictive values but also whether a panel combining these biomarkers could provide more accurate and clinically useful information.

This study has several limitations. First, the single-center design and a limited sample size may restrict the generalizability of our findings, despite the cohort size being comparable to those in other exploratory biomarker studies in kidney transplant populations [35,39]. Future multicenter studies with larger cohorts are needed to validate the predictive utility of urinary ApoA-IV across diverse demographic and clinical settings. In addition, the patient selection process may have introduced bias, as we excluded patients with graft failure, recent acute rejection, and active infections. This exclusion may have resulted in the selection of a healthier subset of KTRs, potentially underestimating the true association between ApoA-IV levels and renal function decline. Patients with severe complications, who were excluded from the study, might have exhibited higher ApoA-IV levels due to their more compromised graft status. To address this limitation, future studies should include a broader range of patients in multicenter cohorts to provide a more comprehensive understanding of the relationship between ApoA-IV levels and renal function across diverse clinical scenarios. Second, not all KTRs in our analysis had kidney biopsies used to make a histologic diagnosis of the CRAI. Third, the lack of serum ApoA-IV measurements is a significant limitation. Without serum levels, we cannot determine whether elevated urinary ApoA-IV reflects increased production, decreased renal clearance, or both. Future studies should include both serum and urinary ApoA-IV measurements to better understand the mechanisms underlying our observations and to potentially improve the predictive value of ApoA-IV as a biomarker. Nevertheless, in addition to examining the cross-sectional relationship between urinary ApoA-IV and renal allograft function, our study also prospectively assessed the significance of urinary ApoA-IV for the progression of renal function deterioration in KTRs. Fourth, another limitation of this study is the focus solely on GFR decline without consideration of proteinuria. Renal function physiologically includes both glomerular filtration and protein excretion. Our study did not assess proteinuria, which is an important marker of kidney damage and can provide additional information about allograft health. Future studies should incorporate both GFR and proteinuria measurements to provide a more comprehensive assessment of renal allograft function in KTRs. Lastly, several factors could potentially confound the relationship between urinary ApoA-IV levels and renal function decline. Immunosuppressive therapy, which all KTRs receive, may affect ApoA-IV metabolism or excretion. However, our study did not assess the impact of different immunosuppressive regimens on ApoA-IV levels. Additionally, factors such as proteinuria, lipid levels, and cardiovascular comorbidities could influence ApoA-IV levels and renal outcomes. Future studies should aim to control for these potential confounders to better elucidate the independent predictive value of urinary ApoA-IV.

In conclusion, our results suggest that urinary ApoA-IV levels might be a potential biomarker for renal allograft function and could be used as a predictor for rapid renal function decline in KTRs. These findings not only highlight the potential utility of ApoA-IV as a non-invasive biomarker but also provide new insights into the molecular mechanisms underlying CRAI. Further studies are warranted to elucidate the specific role of ApoA-IV in transplant outcomes and to explore its potential as a therapeutic target in managing CRAI. In addition, future research should focus on direct comparisons between urinary ApoA-IV and other established biomarkers such as NGAL and KIM-1 to better define its specific role in the clinical management of KTRs.

## Supporting information

S1 File (PDF)
