# Supplementary material for: Urinary apolipoprotein A4 as a biomarker for renal allograft injury in kidney transplant recipients
Source: PLoS One. 2025 May 21;20(5):e0324529. doi: 10.1371/journal.pone.0324529 (PMC12094748; doi:10.1371/journal.pone.0324529)

Supplement 1

Supplement Table 1. Proteins differentially expressed in urine of kidney transplant patients with chronic renal allograft injury versus control group

Supplement Table 2. Simple and multiple linear regression analysis for factors related to annual declines of estimated glomerular filtration rate (mL/min/1.73m^2 per year) in kidney transplant recipients

Supplement Figure 1. Principal component analysis for chronic renal allograft injury (CRAI) versus control group

Supplement Figure 2. Volcano plot for proteins differentially expressed in urine of kidney transplant patients with chronic renal allograft injury (CRAI) versus control group

Supplement Table 1. Proteins differentially expressed in urine of kidney transplant patients with chronic renal allograft injury versus control group

Protein ID	Gene	Log2(Fold Change)	-log(p-value)*
ALBU_HUMAN	ALB	1.68	4.7
Q53H26_HUMAN	Transferrin	2.17	6.0
ZA2G_HUMAN	AZGP1	2.20	2.7
E9KL23_HUMAN	SERPINA1	2.11	4.3
APOA4_HUMAN	APOA4	3.02	2.2
A8K008_HUMAN	A8K008_HUMAN	1.44	1.9
HEMO_HUMAN	HPX	2.38	2.8
E9PEA4_HUMAN	*Uromodulin*	-0.82	1.3
AMBP_HUMAN	AMBP	0.59	0.9
CO3_HUMAN	C3	1.10	2.9
Q5VY30_HUMAN	RBP4	4.43	1.6
Q6N093_HUMAN	DKFZp686I04196	1.38	1.8
Q6PIL8_HUMAN	IGK@	1.69	2.2
APOA1_HUMAN	APOA1	1.66	1.7
A1AG1_HUMAN	ORM1	0.94	0.9
Q96K68_HUMAN	*(SNC73)*	0.31	0.6
Q6NSB4_HUMAN	HP	1.79	2.0
G3V5I3_HUMAN	SERPINA3	1.66	3.0
Q86U78_HUMAN	Angiotensinogen	2.60	3.1
D3DNU8_HUMAN	KNG1	-0.45	0.9
C9JF17_HUMAN	APOD	0.66	0.8
Q8N5F4_HUMAN	IGL@	1.09	1.4
Q53F26_HUMAN	Alpha-amylase	-0.03	0.0
B2MG_HUMAN	B2M	3.08	3.3
B1AVU8_HUMAN	*(PSAP)*	-0.74	2.2
E9PGN5_HUMAN	*ITIH4*	0.94	2.1
F8W7I2_HUMAN	*PTGDS*	-0.95	1.4
Q1L857_HUMAN	Ceruloplasmin	1.67	3.8
C9JV77_HUMAN	AHSG	1.91	4.0
Q68CK4_HUMAN	*(LRG1)*	1.30	1.7
SAP3_HUMAN	GM2A	0.39	0.5
H6VRG2_HUMAN	KRT1	-0.18	0.2

Q59G97_HUMAN	*(ECM1)*	1.36	2.8
B4DVE1_HUMAN	*(Galectin 3)*	-0.49	1.3
Q6EZE9_HUMAN	DEFA3	1.92	0.7
Q5UGI6_HUMAN	SERPING1	1.06	1.2
MASP2_HUMAN	MASP2	0.07	0.1
Q96MH4_HUMAN	Q96MH4_HUMAN	-1.20	2.7
PGBM_HUMAN	HSPG2	1.05	2.0
D6RF35_HUMAN	GC	1.07	2.5
E7EVD2_HUMAN	*EGF*	-1.37	2.6
ANT3_HUMAN	SERPINC1	1.66	4.7
E9KL36_HUMAN	TTR	2.41	1.2
G9JKG7_HUMAN	EPO	0.42	0.9
Q0ZCH9_HUMAN	Q0ZCH9_HUMAN	0.93	1.7
F5H1A8_HUMAN	*GSN*	1.15	1.6
A1BG_HUMAN	A1BG	1.10	1.7
S10A8_HUMAN	S100A8	0.58	0.4
K1C10_HUMAN	KRT10	-0.80	2.5
APOA2_HUMAN	APOA2	0.66	1.9
B4DPC7_HUMAN	B4DPC7_HUMAN	-1.27	2.5
AFAM_HUMAN	AFM	1.03	2.5
PEDF_HUMAN	SERPINF1	3.62	1.4
A1AG2_HUMAN	ORM2	0.58	0.5
J3KN62_HUMAN	*CDH13*	-0.59	1.0
H0YBE2_HUMAN	CA1	3.22	4.6
F1C4A7_HUMAN	CD14	1.17	1.9
A8K7G6_HUMAN	*(REG1A)*	0.09	0.1
PI16_HUMAN	PI16	1.87	3.2
RNAS2_HUMAN	RNASE2	-1.60	4.7
Q9UL78_HUMAN	Q9UL78_HUMAN	1.21	1.8
FIBA_HUMAN	FGA	0.12	0.2
Q53HP2_HUMAN	TPP1	1.20	1.8
B2R4M6_HUMAN	Protein S100	0.05	0.0
D9IWP9_HUMAN	b2GP-1	-0.05	0.1
I3V9T0_HUMAN	CTSC	-0.41	0.6
PIGR_HUMAN	PIGR	0.53	0.7
A2NJV5_HUMAN	IGKV	0.73	0.9
EPCR_HUMAN	PROCR	-0.90	1.9

LYAG_HUMAN	GAA	-0.64	1.6
Q3LGB0_HUMAN	*Osteopontin*	-1.21	4.2
Q6MZM7_HUMAN	DKFZp686O12165	-0.79	2.0
B4DL17_HUMAN	*(KRT13)*	-0.77	0.4
B7Z9W7_HUMAN	*(IBP7)*	-1.23	2.4
F5GZ12_HUMAN	*SPRR3*	-0.66	0.3
A0A5E4_HUMAN	Uncharacterized protein	1.27	2.0
A0N5G5_HUMAN	*RF*	1.27	2.0
B4DV35_HUMAN	*(DNAS1)*	-1.37	3.1
Q9UP81_HUMAN	HBB	0.42	0.3
Q9NP29_HUMAN	*MFAP2*	1.26	2.3
Q9H5A7_HUMAN	*(CD44 antigen)*	-1.45	3.7
Q6T776_HUMAN	KLK1	-1.45	4.4
Q5HYG5_HUMAN	DKFZp686N0152	-0.27	0.3
Q53HQ8_HUMAN	*(Granulin variant)*	-0.75	2.3
E9PBB5_HUMAN	*TFF3*	0.05	0.0
D6RIU4_HUMAN	LMAN2	0.61	1.0
RETN_HUMAN	RETN	0.41	0.6
GPX3_HUMAN	GPX3	1.32	2.6
I3L3X0_HUMAN	ZG16B	-1.37	1.8
B4DVY9_HUMAN	*(Pepsin)*	0.23	0.5
B3KTP7_HUMAN	*(Collagen-a)*	-0.32	0.6
B2R8I2_HUMAN	*(HRG)*	1.26	3.1
B2R9F2_HUMAN	*(SERPINA6)*	1.93	3.8
DPP4_HUMAN	DPP4	0.80	1.8
B7WNR0_HUMAN	(ALB)	0.77	1.3
Q5FBY0_HUMAN	*ACPP*	-2.24	2.0

*by t-test

CRAI, chronic renal allograft injury.

Supplement Table 2. Simple and multiple linear regression analysis for factors related to annual declines of estimated glomerular filtration rate (mL/min/1.73m^2 per year) in kidney transplant recipients

Variables	Simple linear regression			Multiple linear regression [a]		
	Beta	SE	*P* value	Beta	SE	*P* value
Recipient male sex (vs. female)	-1.10	0.93	0.241	-1.10	0.99	0.271
Recipient age	0.05	0.04	0.298	0.002	0.05	0.972
Donor age	0.01	0.03	0.806	-0.03	0.03	0.448
Diabetes mellitus	1.12	1.01	0.271	1.43	1.07	0.187
Acute rejection episodes	0.27	1.10	0.804	0.97	1.16	0.405
Calcineurin inhibitor	0.90	0.94	0.345	0.70	1.00	0.486
Urinary ApoA-IV	-0.01	0.003	0.042	-0.007	0.003	0.036

[a] Adjusted for recipient age, recipient sex, donor age, acute rejection episodes, diabetes mellitus and calcineurin inhibitor.

SE, standard error; ApoA-IV, apolipoprotein A4.

Figure 1. Principal component analysis for chronic renal allograft injury (CRAI) versus control group

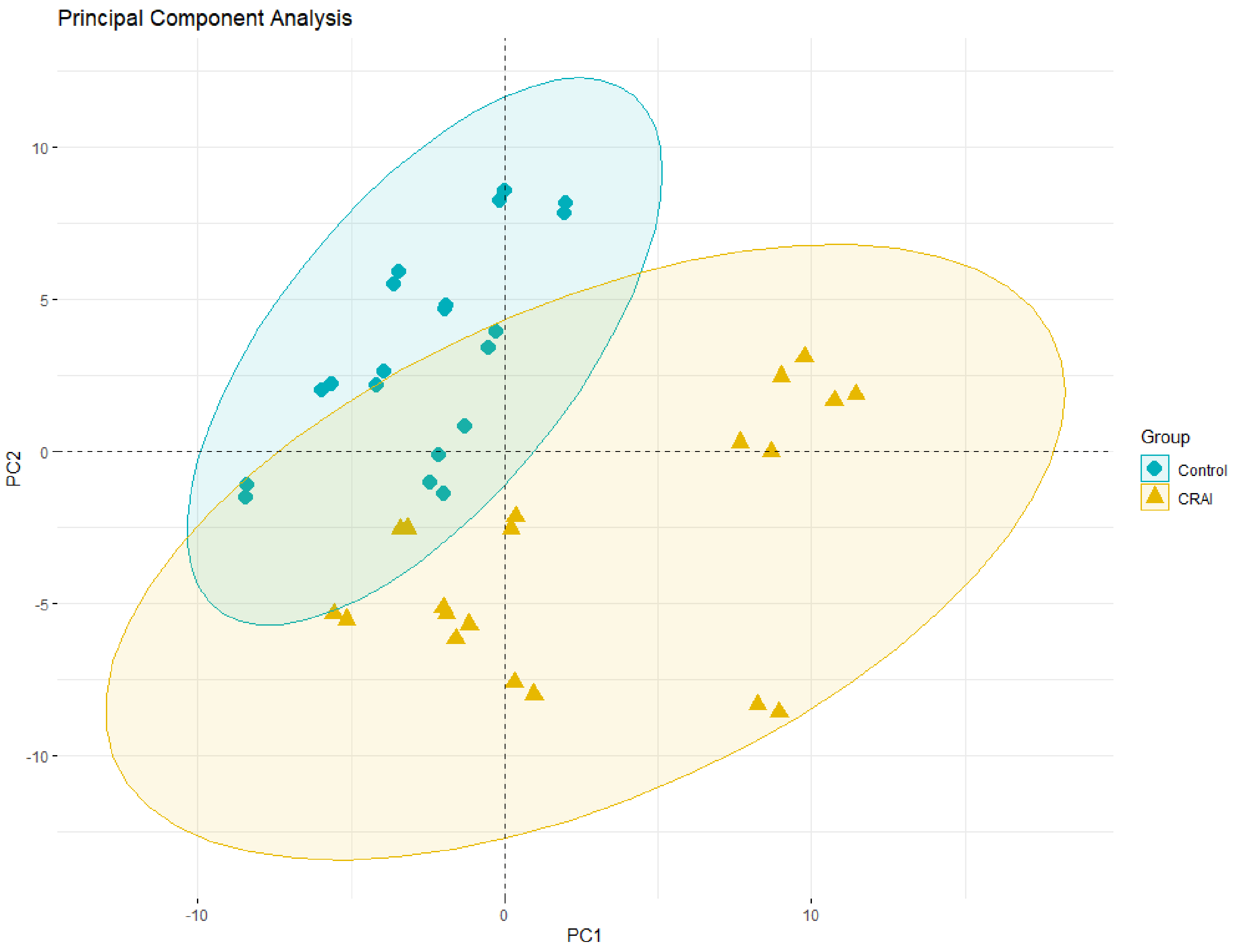

Figure 2. Volcano plot for proteins differentially expressed in urine of kidney transplant patients with chronic renal allograft injury (CRAI) versus control group

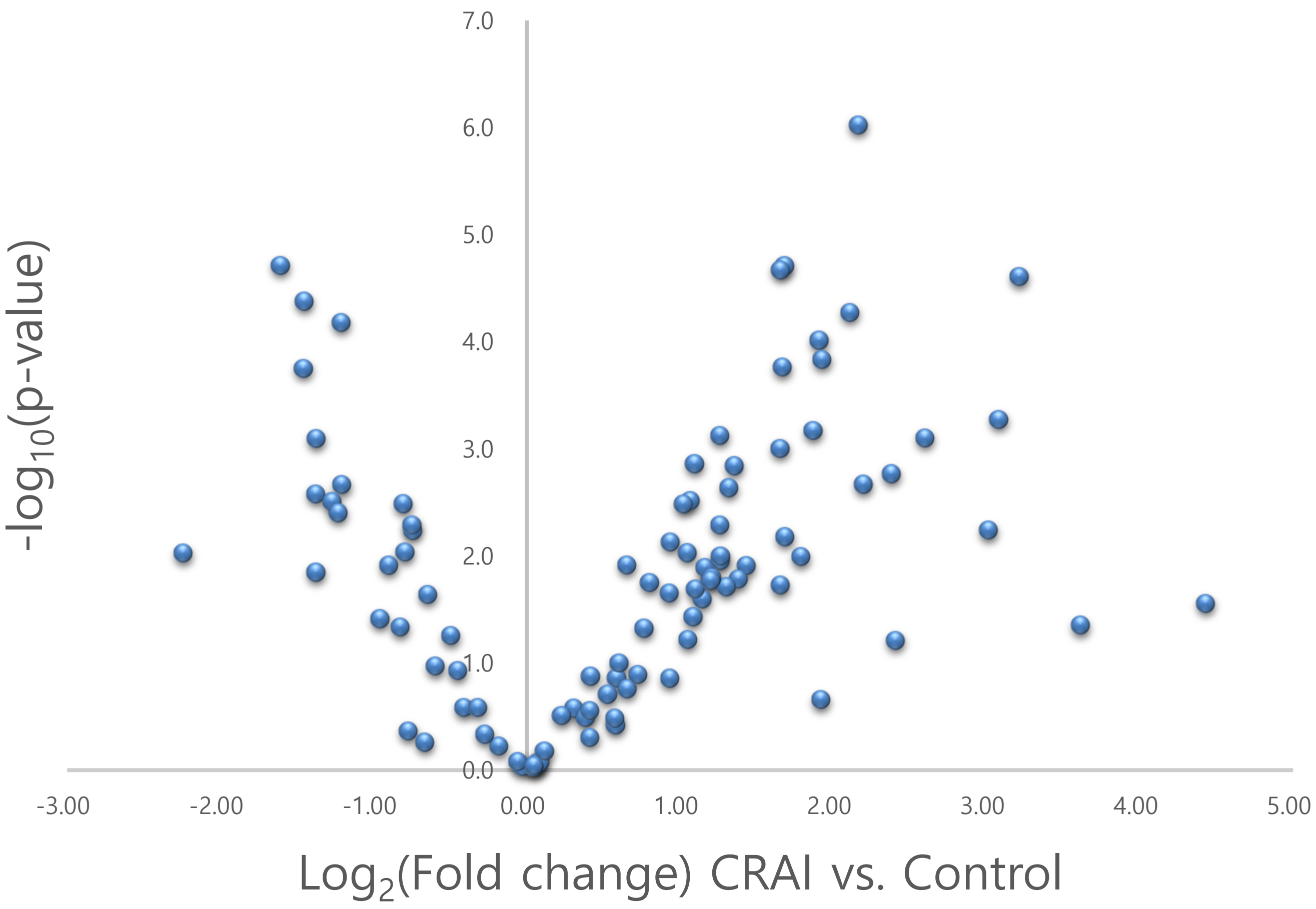

Supplement: S1 File — (PDF) [file pone.0324529.s001.pdf]
